# Representing default knowledge in biomedical ontologies: application to the integration of anatomy and phenotype ontologies

**DOI:** 10.1186/1471-2105-8-377

**Published:** 2007-10-09

**Authors:** Robert Hoehndorf, Frank Loebe, Janet Kelso, Heinrich Herre

**Affiliations:** 1 Department of Evolutionary Genetics, Max Planck Institute for Evolutionary Anthropology, Deutscher Platz 6, 04103 Leipzig, Germany; 2 Institute of Medical Informatics, Statistics and Epidemiology (IMISE), University of Leipzig, Härtelstraße 16-18, 04107 Leipzig, Germany; 3 Institute for Informatics, University of Leipzig, Johannisgasse 26, 04103 Leipzig, Germany

## Abstract

**Background:**

Current efforts within the biomedical ontology community focus on achieving interoperability between various biomedical ontologies that cover a range of diverse domains. Achieving this interoperability will contribute to the creation of a rich knowledge base that can be used for querying, as well as generating and testing novel hypotheses. The OBO Foundry principles, as applied to a number of biomedical ontologies, are designed to facilitate this interoperability. However, semantic extensions are required to meet the OBO Foundry interoperability goals. Inconsistencies may arise when ontologies of properties – mostly phenotype ontologies – are combined with ontologies taking a canonical view of a domain – such as many anatomical ontologies. Currently, there is no support for a correct and consistent integration of such ontologies.

**Results:**

We have developed a methodology for accurately representing canonical domain ontologies within the OBO Foundry. This is achieved by adding an extension to the semantics for relationships in the biomedical ontologies that allows for treating canonical information as default. Conclusions drawn from default knowledge may be revoked when additional information becomes available. We show how this extension can be used to achieve interoperability between ontologies, and further allows for the inclusion of more knowledge within them. We apply the formalism to ontologies of mouse anatomy and mammalian phenotypes in order to demonstrate the approach.

**Conclusion:**

Biomedical ontologies require a new class of relations that can be used in conjunction with default knowledge, thereby extending those currently in use. The inclusion of default knowledge is necessary in order to ensure interoperability between ontologies.

## Background

As the volume of biomedical data and knowledge presented in scientific papers increases, there is an increasing need to support formal analyses of these data and to pre-process knowledge for further use in solving problems and developing and testing hypotheses. The precise capture of biological data and knowledge and their correct and consistent representation in computational form is a basic pre-requisite for achieving these goals. Ontologies may provide a basis for integrating, processing and applying biomedical data. Their integration into a common ontological framework is an indispensible step towards the development of expressive knowledge bases. Interoperability between these ontologies would facilitate the consistent use of biomedical data in the form of annotations, allow for queries over multiple ontologies and form a rich knowledge resource for biomedicine that could be further used in solving problems and stating hypotheses. Different ontologies have been developed by different groups with different intentions. As a result, translating a statement or transferring an annotation from one ontology to another may not always yield the correct results. The absence of clear principles for achieving interoperability between different ontologies hinders the development of advanced applications and analysis tools based on these ontologies. A number of biomedical ontologies exist, which cover domains such as anatomy [1], cell structure, biological processes, functions [2], diseases [3], development [4], experimental conditions, phenotypes, qualities [5] and relationships [6]. A subset of these are unified under the umbrella of the Open Biomedical Ontologies (OBO) Foundry [7]. The OBO Relationship Ontology [6], together with the principles set forth in the OBO Foundry [8] have contributed to better interoperability between a large number of these ontologies. We address here several remaining problems.

One particular difficulty in making these ontologies interoperable results from the existence of two particular types of biomedical ontologies. The first group describes a *canonical *or idealized view on a domain, such as an ontology of canonical anatomy. The other group describes *phenotypes*, properties or phenomena, that – when exemplified by individuals – may contradict knowledge represented in the first group. We call the former group *canonical ontologies *and the latter *phenotype ontologies*. An example of a canonical ontology is the Foundational Model of Anatomy [9] (FMA), which describes an idealized domain, i.e., it describes a prototypical, idealized human anatomy. Many ontologies describing structure, such as cell structure, histology or anatomy, are canonical in this sense. On the other hand, a phenotype ontology describes phenomena whose exemplification by individuals may lead to deviations from this idealized structure. For example, the Mammalian Phenotype Ontology [10] contains the term "absent tail" as a specific type of "abnormal tail morphology". When a researcher would like to refer to an individual mouse with an "absent tail", this mouse does not comply with the canonical, idealized mouse anatomy that excludes such abnormalities.

The integration of ontologies of these different types cannot be achieved using methods developed hitherto, and a new set of methods transcending the framework of classical logic must be introduced to avoid inconsistencies while preserving the specificity of both types of knowledge. We present an approach that uses nonmonotonic reasoning to integrate canonical and phenotype ontologies.

## Methods and Results

### GFO-Bio

Integrating ontologies is a powerful means for achieving interoperability. We adopt John Sowa's definition of ontology integration [[11], p. 494], which he characterized as the process of finding commonalities between different ontologies *A *and *B *and deriving a new, integrated ontology *C *that facilitates interoperability between information systems based on ontologies *A *and *B*. There are several approaches to achieving such an integration [12], but there is no generally accepted solution.

**Figure 1:**
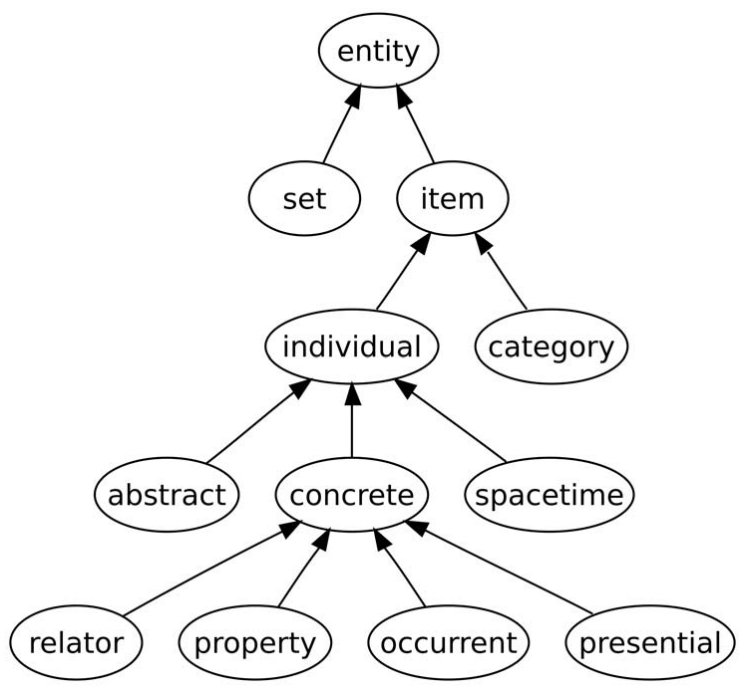
The main categories of the General Formal Ontology (GFO) as outlined in [14, p. 70]. The basic distinction of GFO relevant for this work relates to items and is between individuals and categories. Individuals are items that cannot be instantiated. Categories are items that can have instances and may be predicated of other entities. The instances of categories of first order are individuals, while higher-order categories have categories as instances. For all items, the **instance-of **relationship is a relation of major importance, linking items (including categories) to the categories of which they are an instance.

Our approach to integration is based on top-level ontologies [13]. For our study, we use the top-level ontology General Formal Ontology (GFO) [14]. GFO has several features that distinguish it from other top-level ontologies such as BFO [15,16] and DOLCE [17]. Among the relevant features are the inclusion of a theory of levels of reality [18], and the explicit incorporation of an ontological theory of higher-order categories (see figure 1 for an overview of selected categories and an explanation of higher-order categories). We have developed GFO-Bio [19], a core ontology [20] for biology. It is formalized in the Web Ontology Language [21] (OWL) and includes aspects of faceted classification [22] combined with GFO's theory of ontological levels of reality [18].

**Figure 2:**
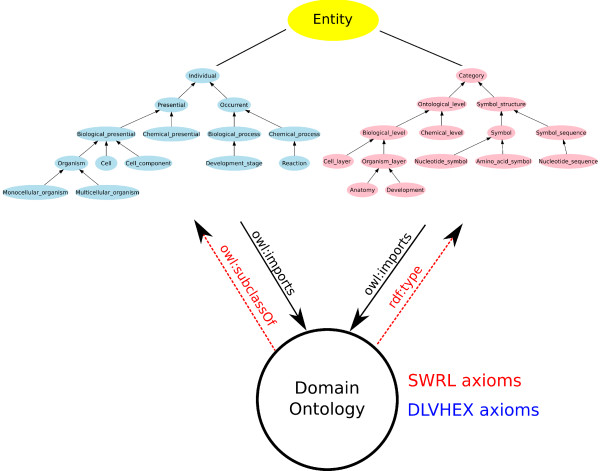
The modules of the biological core ontology GFO-Bio. GFO-Bio comes in two modules. The first focuses on individuals and defines categories of individuals like "Cell" or "Organism" in the Web Ontology Language (OWL). It contains 168 categories and 73 relations. The second treats categories as instances in OWL and describes interrelations between domains or whole domain ontologies. It is based on a theory of levels of reality and contains 43 categories and two additional relations. Both modules are related using the Semantic Web Rule Language [44] and DLVHEX rules.

GFO-Bio comprises an ontology of individuals, similar to other established upper biomedical ontologies. "Biological individual" is introduced as a subclass of GFO's "Individual" category. The classes are defined or restricted using description logic statements. For example, a "Molecule" is a subclass of "Material object", which has as part at least two atoms. In addition, GFO-Bio contains another branch, in which categories themselves are further described and defined. This is an ontology of categories within the biomedical domain. It is this part of GFO-Bio that can directly represent directed acyclic graphs, which are commonly used for many biomedical ontologies. For an overview of the modules of GFO-Bio, see figure 2. In the remainder of this section, the basic ontology used can be considered to contain only two categories, "Individual" and "Category". We prefix relationships between categories with *CC *and relationships between individuals with *II*. Relationships between categories and individuals are prefixed with *CI *or *IC *respectively. For example, the relationship **IC-instance-of **is the instantiation relation, and the relation **CC-isa **is the is-a relation.

### Default rules and default logic

Using GFO-Bio as a framework for integrating biomedical ontologies, we address the problem of accurately representing canonical and phenotype ontologies. A canonical anatomy ontology such as the Foundational Model of Anatomy [9] (FMA) establishes rules such as:

Every instance of a human body has as part an appendix.

This does not necessarily apply to every real human body: an individual human body may *lack *an appendix as part. However, the rule describes an idealized or *canonical *human. Phenotype ontologies describe phenomena, whose exemplification by individuals can be *deviations *from these idealizations. For example, an individual may be both an instance of a prototypical human body as described in the FMA (which implies an appendix as part) and an instance of the category "human body with absent appendix". In a classical logical framework, such as those commonly used in biomedical knowledge representation, e.g. in the form of OWL [21], a formalization of the conjunction of these two statements would lead to an inconsistency. A human body in the former case has an appendix as a part, while in the latter case it does not. Instantiating both categories creates the inconsistency. A logical inconsistency in the formal sense can only arise when the logical functor of negation is used. This functor is hidden in concepts such as "absent X", as used in the Mammalian Phenotype Ontology [10]. The formal detection of logical inconsistencies by inferences needs an explication of negation.

In order to avoid terms such as "absent X" and make the negation explicit, we adopt a modified form of the **lacks **relation [23], which we explicitly define as:

Individual *p ***lacks **category *C *with respect to relation **R**, if and only if there does not exist an *x *such that: *p***R***x *and *x *is an instance of *C*.

We use binary relations of the kind *x ***lacks-R ***C *instead of *x ***lacks ***C *with respect to **R**. For example, the fact that some individual *x ***lacks **a category *C *with respect to the relation **has-part **will be denoted as *x ***lacks-part ***C*.

Using the **lacks **relation may cause an inconsistency when a canonical ontology and a corresponding phenotype ontology are used in a classical logic formalism, such as first order logic [24] or description logic [25]. The reason is that classical formalisms enforce very strict interpretations, e.g. of quantifications like "every human", which results in *monotonicity *of these formalisms: the inferences drawn from a classical logical theory *T *remain true in every extension of *T *with additional facts.

In order to prevent inconsistencies, while at the same time preserving the intuition behind statements such as "a human has an appendix as part", the interpretation of such statements in the canonical ontology must be modified. We propose to use a *nonmonotonic *logic that treats the statements provided in a canonical ontology as true by default. Adding further knowledge, e.g. by referring to a phenotype ontology or using a statement involving the **lacks **relation (and therefore negation), may invalidate previously drawn conclusions.

Several ways of treating default rules and exceptions in logics have been proposed. The most popular among these proposals are default logic [26], circumscription [27,28] and autoepistemic logic [29,30]. We use default logic for our application, because it admits a transparent representation, and allows a semantically correct translation to a form of nonmonotonic, declarative logic programs called answer set programs [31].

In default logic, a *default rule *has the following form:

A(x¯):B(x¯)C(x¯)
 MathType@MTEF@5@5@+=feaafiart1ev1aaatCvAUfKttLearuWrP9MDH5MBPbIqV92AaeXatLxBI9gBaebbnrfifHhDYfgasaacH8akY=wiFfYdH8Gipec8Eeeu0xXdbba9frFj0=OqFfea0dXdd9vqai=hGuQ8kuc9pgc9s8qqaq=dirpe0xb9q8qiLsFr0=vr0=vr0dc8meaabaqaciaacaGaaeqabaqabeGadaaakeaadaWcaaqaaiabdgeabjabcIcaOiqbdIha4zaaraGaeiykaKIaeiOoaOJaemOqaiKaeiikaGIafmiEaGNbaebacqGGPaqkaeaacqWGdbWqcqGGOaakcuWG4baEgaqeaiabcMcaPaaaaaa@3AA8@

This means that if *A*(x¯
 MathType@MTEF@5@5@+=feaafiart1ev1aaatCvAUfKttLearuWrP9MDH5MBPbIqV92AaeXatLxBI9gBaebbnrfifHhDYfgasaacH8akY=wiFfYdH8Gipec8Eeeu0xXdbba9frFj0=OqFfea0dXdd9vqai=hGuQ8kuc9pgc9s8qqaq=dirpe0xb9q8qiLsFr0=vr0=vr0dc8meaabaqaciaacaGaaeqabaqabeGadaaakeaacuWG4baEgaqeaaaa@2E3D@) is true (prerequisite), and *it is consistent to assume *that *B*(x¯
 MathType@MTEF@5@5@+=feaafiart1ev1aaatCvAUfKttLearuWrP9MDH5MBPbIqV92AaeXatLxBI9gBaebbnrfifHhDYfgasaacH8akY=wiFfYdH8Gipec8Eeeu0xXdbba9frFj0=OqFfea0dXdd9vqai=hGuQ8kuc9pgc9s8qqaq=dirpe0xb9q8qiLsFr0=vr0=vr0dc8meaabaqaciaacaGaaeqabaqabeGadaaakeaacuWG4baEgaqeaaaa@2E3D@), then *C*(x¯
 MathType@MTEF@5@5@+=feaafiart1ev1aaatCvAUfKttLearuWrP9MDH5MBPbIqV92AaeXatLxBI9gBaebbnrfifHhDYfgasaacH8akY=wiFfYdH8Gipec8Eeeu0xXdbba9frFj0=OqFfea0dXdd9vqai=hGuQ8kuc9pgc9s8qqaq=dirpe0xb9q8qiLsFr0=vr0=vr0dc8meaabaqaciaacaGaaeqabaqabeGadaaakeaacuWG4baEgaqeaaaa@2E3D@) can be derived. Intuitively, *A*(x¯
 MathType@MTEF@5@5@+=feaafiart1ev1aaatCvAUfKttLearuWrP9MDH5MBPbIqV92AaeXatLxBI9gBaebbnrfifHhDYfgasaacH8akY=wiFfYdH8Gipec8Eeeu0xXdbba9frFj0=OqFfea0dXdd9vqai=hGuQ8kuc9pgc9s8qqaq=dirpe0xb9q8qiLsFr0=vr0=vr0dc8meaabaqaciaacaGaaeqabaqabeGadaaakeaacuWG4baEgaqeaaaa@2E3D@) is a prerequisite, and assuming *B*(x¯
 MathType@MTEF@5@5@+=feaafiart1ev1aaatCvAUfKttLearuWrP9MDH5MBPbIqV92AaeXatLxBI9gBaebbnrfifHhDYfgasaacH8akY=wiFfYdH8Gipec8Eeeu0xXdbba9frFj0=OqFfea0dXdd9vqai=hGuQ8kuc9pgc9s8qqaq=dirpe0xb9q8qiLsFr0=vr0=vr0dc8meaabaqaciaacaGaaeqabaqabeGadaaakeaacuWG4baEgaqeaaaa@2E3D@) adds justification for deriving *C*(x¯
 MathType@MTEF@5@5@+=feaafiart1ev1aaatCvAUfKttLearuWrP9MDH5MBPbIqV92AaeXatLxBI9gBaebbnrfifHhDYfgasaacH8akY=wiFfYdH8Gipec8Eeeu0xXdbba9frFj0=OqFfea0dXdd9vqai=hGuQ8kuc9pgc9s8qqaq=dirpe0xb9q8qiLsFr0=vr0=vr0dc8meaabaqaciaacaGaaeqabaqabeGadaaakeaacuWG4baEgaqeaaaa@2E3D@) from *A*(x¯
 MathType@MTEF@5@5@+=feaafiart1ev1aaatCvAUfKttLearuWrP9MDH5MBPbIqV92AaeXatLxBI9gBaebbnrfifHhDYfgasaacH8akY=wiFfYdH8Gipec8Eeeu0xXdbba9frFj0=OqFfea0dXdd9vqai=hGuQ8kuc9pgc9s8qqaq=dirpe0xb9q8qiLsFr0=vr0=vr0dc8meaabaqaciaacaGaaeqabaqabeGadaaakeaacuWG4baEgaqeaaaa@2E3D@). Thus, as long as *B*(x¯
 MathType@MTEF@5@5@+=feaafiart1ev1aaatCvAUfKttLearuWrP9MDH5MBPbIqV92AaeXatLxBI9gBaebbnrfifHhDYfgasaacH8akY=wiFfYdH8Gipec8Eeeu0xXdbba9frFj0=OqFfea0dXdd9vqai=hGuQ8kuc9pgc9s8qqaq=dirpe0xb9q8qiLsFr0=vr0=vr0dc8meaabaqaciaacaGaaeqabaqabeGadaaakeaacuWG4baEgaqeaaaa@2E3D@) can be assumed, default logic concludes *C*(x¯
 MathType@MTEF@5@5@+=feaafiart1ev1aaatCvAUfKttLearuWrP9MDH5MBPbIqV92AaeXatLxBI9gBaebbnrfifHhDYfgasaacH8akY=wiFfYdH8Gipec8Eeeu0xXdbba9frFj0=OqFfea0dXdd9vqai=hGuQ8kuc9pgc9s8qqaq=dirpe0xb9q8qiLsFr0=vr0=vr0dc8meaabaqaciaacaGaaeqabaqabeGadaaakeaacuWG4baEgaqeaaaa@2E3D@). In order to formalize our example of humans normally having an appendix as part, we would use the following default rule:

Human(x):x IC-has-part Appendixx IC-has-part Appendix
 MathType@MTEF@5@5@+=feaafiart1ev1aaatCvAUfKttLearuWrP9MDH5MBPbIqV92AaeXatLxBI9gBaebbnrfifHhDYfgasaacH8akY=wiFfYdH8Gipec8Eeeu0xXdbba9frFj0=OqFfea0dXdd9vqai=hGuQ8kuc9pgc9s8qqaq=dirpe0xb9q8qiLsFr0=vr0=vr0dc8meaabaqaciaacaGaaeqabaqabeGadaaakeaadaWcaaqaaiabdIeaijabdwha1jabd2gaTjabdggaHjabd6gaUjabcIcaOiabdIha4jabcMcaPiabcQda6iabdIha4jabbccaGGqabiab=Leajjab=neadjabb2caTiab=HgaOjab=fgaHjab=nhaZjabb2caTiab=bhaWjab=fgaHjab=jhaYjab=rha0jabbccaGiabdgeabjabdchaWjabdchaWjabdwgaLjabd6gaUjabdsgaKjabdMgaPjabdIha4bqaaiabdIha4jabbccaGiab=Leajjab=neadjabb2caTiab=HgaOjab=fgaHjab=nhaZjabb2caTiab=bhaWjab=fgaHjab=jhaYjab=rha0jabbccaGiabdgeabjabdchaWjabdchaWjabdwgaLjabd6gaUjabdsgaKjabdMgaPjabdIha4baaaaa@6DE8@

Here, the precondition is *Human*(*x*), the fact that *x *is a human. Then, if it is consistent to assume that *x *has as part an instance of *Appendix*, it is concluded that *x *has as part an instance of *Appendix*. The definition of the relation **IC-has-part **follows the schema in table 1.

**Table 1:** Schema of introduced relations

**Relation**	**Domain:Range**	**Definition**
*x ***II-R ***y*	Individual:Individual	The individuals *x *and *y *stand in the relationship **II-R**.
*x ***IC-R ***y*	Individual:Category	There exists an individual *z*, such that: *z ***IC-instance-of ***y *and *x ***II-R ***z*.
*x ***CC-R ***y*	Category:Category	For all individuals *a *such that: *a ***IC-instance-of ***x*, *a ***IC-R ***y*.
*x ***CC-canonical-R ***y*	Category:Category	For all individuals *a *such that: *a ***IC-instance-of ***x*, by default, *a ***IC-R ***y*.
*x ***II-lacks-R ***y*	Individual:Individual	The individuals *x *and *y *do not stand in the relationship **II-R**.
*x ***IC-lacks-R ***y*	Individual:Category	The individual *x *does not stand in the relationship **IC-R **to *y*.
*x ***CC-lacks-R ***y*	Category:Category	For all individuals *a *such that: *a ***IC-instance-of ***x*, *a ***IC-lacks-R ***y*.

For each relation used in a biomedical ontology, a number of relations between categories, individuals and between individuals and categories can be created. The **CC-canonical-R **relationship is a *default *relation that is accompanied by axioms in an answer set program to describe its semantics as a default.

Nonmonotonicity arises from "it is consistent to assume that *x ***IC-has-part ***Appendix*", which means that if *x ***IC-has-part ***Appendix *cannot be proven false from the given facts, its addition to the knowledge base does not lead to a contradiction. Adding the statement that *x *does not have an appendix as part (*x ***IC-lacks-part ***Appendix*) would lead to an inconsistency with *x ***IC-has-part ***Appendix*; therefore, this rule could no longer be used to derive that *x *has an appendix as part.

Answer-set programming, the formalism we use for our implementation, can mimic default rules. It uses two kinds of negation, called *strong *and *weak negation*. Strong negation is the classical (monotonic) negation, as used in the definition of the **lacks **relation. Weak negation, often denoted as not A, corresponds to the above statements "it cannot be proven that A is true", or "it is consistent to assume that A is false".

### Formalizing defaults in biomedical ontologies

In a canonical ontology, relationships between its categories can be interpreted as *default *relations. By default, a human has some appendix as part. However, an instance of a human, such as *John*, may **lack **an appendix as a part; therefore, *John *is an instance of both "human" and "human without an appendix" (or "absent appendix"). In order to include canonical relationships between two categories, new relations must be introduced, such as **CC-canonical-has-part**. Then, the relationship between "human" and "appendix" becomes "human **CC-canonical-has-part **appendix". Further, this relationship corresponds to a *default rule*:

forall x,C1,C2:if C1 CC-canonical-has-part C2 and x IC-instance-of C1,thenby default:there exists a y:y IC-instance-of C2 and x II-has-party
 MathType@MTEF@5@5@+=feaafiart1ev1aaatCvAUfKttLearuWrP9MDH5MBPbIqV92AaeXatLxBI9gBaebbnrfifHhDYfgasaacH8akY=wiFfYdH8Gipec8Eeeu0xXdbba9frFj0=OqFfea0dXdd9vqai=hGuQ8kuc9pgc9s8qqaq=dirpe0xb9q8qiLsFr0=vr0=vr0dc8meaabaqaciaacaGaaeqabaqabeGadaaakeGabqacbuaabaqaeeaaaaqaaiabbAgaMjabb+gaVjabbkhaYjabbggaHjabbYgaSjabbYgaSjabbccaGiabdIha4jabcYcaSiabdoeadnaaBaaaleaacqaIXaqmaeqaaOGaeiilaWIaem4qam0aaSbaaSqaaiabikdaYaqabaGccqGG6aGoaeGacmaDbmasdiaaxMaacqqGPbqAcqqGMbGzcqqGGaaicqWGdbWqdaWgaaWcbaGaeGymaedabeaakiabbccaGGqabiab=neadjab=neadjabb2caTiab=ngaJjab=fgaHjab=5gaUjab=9gaVjab=5gaUjab=LgaPjab=ngaJjab=fgaHjab=XgaSjabb2caTiab=HgaOjab=fgaHjab=nhaZjabb2caTiab=bhaWjab=fgaHjab=jhaYjab=rha0jabbccaGiabdoeadnaaBaaaleaacqaIYaGmaeqaaOGaeeiiaaIaeeyyaeMaeeOBa4MaeeizaqMaeeiiaaIaemiEaGNaeeiiaaIae8xsaKKae83qamKaeeyla0Iae8xAaKMae8NBa4Mae83CamNae8hDaqNae8xyaeMae8NBa4Mae83yamMae8xzauMaeeyla0Iae83Ba8Mae8NzayMaeeiiaaIaem4qam0aaSbaaSqaaiabigdaXaqabaGccqGGSaalcqqG0baDcqqGObaAcqqGLbqzcqqGUbGBaeGafiafhiauhia+hiadjiasjiaaxMaacqqGIbGycqqG5bqEcqqGGaaicqqGKbazcqqGLbqzcqqGMbGzcqqGHbqycqqG1bqDcqqGSbaBcqqG0baDcqGG6aGoaeGabqaJbiaaxMaacqqG0baDcqqGObaAcqqGLbqzcqqGYbGCcqqGLbqzcqqGGaaicqqGLbqzcqqG4baEcqqGPbqAcqqGZbWCcqqG0baDcqqGZbWCcqqGGaaicqqGHbqycqqGGaaicqWG5bqEcqGG6aGocqWG5bqEcqqGGaaicqWFjbqscqWFdbWqcqqGTaqlcqWFPbqAcqWFUbGBcqWFZbWCcqWF0baDcqWFHbqycqWFUbGBcqWFJbWycqWFLbqzcqqGTaqlcqWFVbWBcqWFMbGzcqqGGaaicqWGdbWqdaWgaaWcbaGaeGOmaidabeaakiabbccaGiabbggaHjabb6gaUjabbsgaKjabbccaGiabdIha4jabbccaGiab=Leajjab=Leajjabb2caTiab=HgaOjab=fgaHjab=nhaZjabb2caTiab=bhaWjab=fgaHjab=jhaYjab=rha0jab=bcaGGqaciab+Lha5baaaaa@DB4F@

Using a class of **lacks **relationships as introduced by [23], we formalize the default operator in the rule above as:

forall x,C1,C2:if C1 CC-canonical-has-part C2 and x IC-instance-of C1 andit cannot be proven that x IC-lacks-part C2,thenthere exists a y:y IC-instance-of C2 and x II-has-part y
 MathType@MTEF@5@5@+=feaafiart1ev1aaatCvAUfKttLearuWrP9MDH5MBPbIqV92AaeXatLxBI9gBaebbnrfifHhDYfgasaacH8akY=wiFfYdH8Gipec8Eeeu0xXdbba9frFj0=OqFfea0dXdd9vqai=hGuQ8kuc9pgc9s8qqaq=dirpe0xb9q8qiLsFr0=vr0=vr0dc8meaabaqaciaacaGaaeqabaqabeGadaaakeaafaqaaeabbaaaaeaacqqGMbGzcqqGVbWBcqqGYbGCcqqGHbqycqqGSbaBcqqGSbaBcqqGGaaicqWG4baEcqGGSaalcqWGdbWqdaWgaaWcbaGaeGymaedabeaakiabcYcaSiabdoeadnaaBaaaleaacqaIYaGmaeqaaOGaeiOoaOdabiqaaayacaWLjaGaeeyAaKMaeeOzayMaeeiiaaIaem4qam0aaSbaaSqaaiabigdaXaqabaGccqqGGaaiieqacqWFdbWqcqWFdbWqcqqGTaqlcqWFJbWycqWFHbqycqWFUbGBcqWFVbWBcqWFUbGBcqWFPbqAcqWFJbWycqWFHbqycqWFSbaBcqqGTaqlcqWFObaAcqWFHbqycqWFZbWCcqqGTaqlcqWFWbaCcqWFHbqycqWFYbGCcqWF0baDcqqGGaaicqWGdbWqdaWgaaWcbaGaeGOmaidabeaakiabbccaGiabbggaHjabb6gaUjabbsgaKjabbccaGiabdIha4jabbccaGiab=Leajjab=neadjabb2caTiab=LgaPjab=5gaUjab=nhaZjab=rha0jab=fgaHjab=5gaUjab=ngaJjab=vgaLjabb2caTiab=9gaVjab=zgaMjabbccaGiabdoeadnaaBaaaleaacqaIXaqmaeqaaOGaeeiiaaIaeeyyaeMaeeOBa4MaeeizaqgabiqaaGOacaWLjaGaeeyAaKMaeeiDaqNaeeiiaaIaee4yamMaeeyyaeMaeeOBa4MaeeOBa4Maee4Ba8MaeeiDaqNaeeiiaaIaeeOyaiMaeeyzauMaeeiiaaIaeeiCaaNaeeOCaiNaee4Ba8MaeeODayNaeeyzauMaeeOBa4MaeeiiaaIaeeiDaqNaeeiAaGMaeeyyaeMaeeiDaqNaeeiiaaIaemiEaGNaeeiiaaIae8xsaKKae83qamKaeeyla0Iae8hBaWMae8xyaeMae83yamMae83AaSMae83CamNaeeyla0Iae8hCaaNae8xyaeMae8NCaiNae8hDaqNaeeiiaaIaem4qam0aaSbaaSqaaiabikdaYaqabaGccqGGSaalcqqG0baDcqqGObaAcqqGLbqzcqqGUbGBaeGabaaPbiaaxMaacqqG0baDcqqGObaAcqqGLbqzcqqGYbGCcqqGLbqzcqqGGaaicqqGLbqzcqqG4baEcqqGPbqAcqqGZbWCcqqG0baDcqqGZbWCcqqGGaaicqqGHbqycqqGGaaicqWG5bqEcqGG6aGocqWG5bqEcqqGGaaicqWFjbqscqWFdbWqcqqGTaqlcqWFPbqAcqWFUbGBcqWFZbWCcqWF0baDcqWFHbqycqWFUbGBcqWFJbWycqWFLbqzcqqGTaqlcqWFVbWBcqWFMbGzcqqGGaaicqWGdbWqdaWgaaWcbaGaeGOmaidabeaakiabbccaGiabbggaHjabb6gaUjabbsgaKjabbccaGiabdIha4jabbccaGiab=Leajjab=Leajjabb2caTiab=HgaOjab=fgaHjab=nhaZjabb2caTiab=bhaWjab=fgaHjab=jhaYjab=rha0jabbccaGiabdMha5baaaaa@051A@

In general, for each relation **R **between the categories in an ontology, we create several new relations: **CC-R **for the monotonic relationship between the categories, **CC-canonical-R **for the nonmonotonic default relationship between categories, **IC-R **for the monotonic relationship between an individual and a category, such as "John **IC-has-part **Appendix", meaning that John has some appendix as part, and **II-R **for the monotonic relationship between individuals. In addition, we introduce a class of **lacks **relationships. A schematic view of the new relationships introduced is shown in table 1. The schema is somewhat incomplete, because the introduction of canonical relations can be extended to the class of **lacks **relation, in the sense that some category may canonically lack some other category with respect to a relation **R**. In this case, the relation **R **must be replaced by **lacks-R**. This allows the treatment of exceptions between categories. For example, the category "Mouse with absent tail" can be defined as a mouse which lacks a tail as part.

### Implementation

We have used a technique known as DL-programs [32] to implement rules together with the OWL version of GFO-Bio. The system DLVHEX allows for a bidirectional flow of information between an answer-set program and a description logic knowledge base or ontology; thus, it is well suited for our purposes. DLVHEX is based on the well-established datalog system DLV [33].

Relationships that are used in GFO-Bio are made available in the DLVHEX system. It then becomes possible to express the necessary axioms for relations of the kind **CC-canonical-R**. For example, for the relationship **CC-canonical-has-part**, the following axiom is added, corresponding to formula (5) in DLVHEX:

IC-has-part(X,Y) :- ind(X),class(Y),class(Z),inst(X,Z),

   CC-canonical-has-part(Z,Y),

   not IC-lacks-part(X,Y).

**Figure 3:**
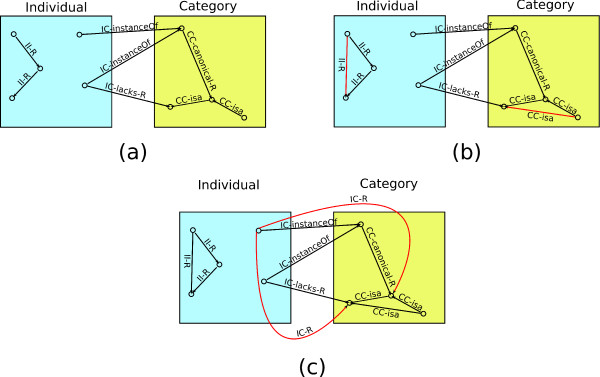
In figure (a), the left side shows five individuals (instances of GFO-Bio's "Individual" category) and the right side contains four categories (instances of GFO-Bio's "Category" category). In addition, a number of relations are illustrated between the individuals, between the categories, and between individuals and categories. The relation **R**, denoted as **II-R**, is transitive. Figure (a) and the transitivity of **II-R **should be seen as the input ontology. In figure (b), the result of a classification using a description logic reasoner is illustrated. Here, the transitivity of the **CC-isa **relation and the relation **II-R **is resolved, reflected by the additional links. Figure (c) shows the result from applying the answer set rules formulated in DLVHEX. In this step, the default relationship between two categories, denoted by **CC-canonical-R**, is resolved. Two additional **IC-R **links are created for one individual. For the other individual, which instantiates the same category, these links are not created, because the **IC-lacks-R **relation blocks them.

This means that if two categories *Z *and *Y *stand in the relation **CC-canonical-has-part**, and *it cannot be proven that X ***IC-lacks-part ***Y *(not IC-lacksPart(X,Y)), then it is concluded that an individual *X*, which is an instance of *Z*, stands in the relation **IC-has-part **to the category *Y*. A simple example illustrating this reasoning is shown in figure 3.

**Figure 4:**
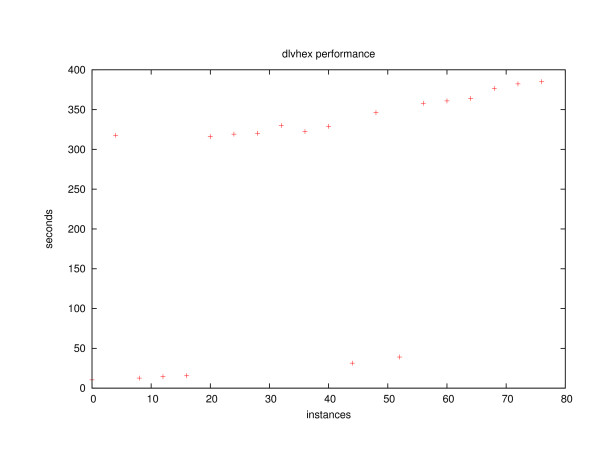
This test uses the Adult Mouse Anatomy Ontology with 2740 classes. For testing, a fixed number of instances of *Adult mouse *is generated for a single run. Moreover, each of the mouse instances is assigned a single exception by means of a **lacks-part ***tail *statement. Using this dataset, a query for all parts of each mouse is run, which yields 2729 parts per mouse (derived from applicable canonical relationships). As intended, tails are not contained, and neither are any parts of tail like *caudal vertebra*. The graph shows the number of seconds for test runs with 0 to 80 mouse instances. For some numbers of instances, the time consumption is considerably less than for others. The difference of over 300 seconds is caused by the answer set solver DLV. We have been unable to examine the source code in order to determine the reason for this behaviour. The dataset used for this test can be accessed from the project webpage [45].

A plot showing the performance of our implementation for a common type of query on a mid-sized ontology is shown in figure 4. The sample test indicates that queries can be answered, but require several minutes. While this may be insufficient in practice for some applications, we believe that it shows that our implementation works, but needs further improvement. An extensive performance evaluation of the proposed method after some improvements on the implementation is subject to future work.

### Ontology integration with GFO-Bio

Integrating biological ontologies using GFO-Bio involves several steps. First, an OWL-DL version of each ontology must be aquired or produced. OWL-DL is a sufficiently expressive language because negation is available and logical inconsistencies can be formally detected in the OWL-DL framework. For the purpose of this conversion, we provide a tool [19] that converts OBO format files [34] into OWL-DL. This conversion yields correct results for ontologies that are built according to the OBO Foundry principles, but may provide incorrect conversions for other ontologies available in the OBO format. The generated OWL-DL file must then be imported by GFO-Bio. Each top-level class of the imported ontology is then defined, at least partially, using categories from GFO-Bio's individual tree. For example, the "Cell" category of the Celltype Ontology [35] must be declared a subclass of (or an equivalent of) GFO-Bio's "Cell" category. Further, a second OWL-DL file can be produced for each integrated ontology containing the ontology's categories as instances of GFO-Bio's category branch. We also provide a tool for performing this conversion for OBO files. This file must be imported by GFO-Bio as well. In this file, relationships between categories, as directly expressed in the OBO-style directed acyclic graphs (DAGs), are modelled as relationships between OWL instances.

For example, the relationship expressed in the DAG of the Gene Ontology's cellular component ontology, "Membrane **part-of **Cell", is represented twice in GFO-Bio: First, "Membrane" and "Cell" are created as classes in OWL, and the following restriction created (in line with [36]):

SubClassOf(Membrane restriction(II-part-of someValuesFrom(Cell)))

In addition, the Gene Ontology's "Cell" category is declared equivalent to GFO-Bio's "Cell" category. Second, "Membrane" and "Cell" are treated as instances of GFO-Bio's "Category" class, and a relation **CC-part-of **('CC' indicating the category-category reading of the relation) between "Membrane" and "Cell" is asserted:

Individual(Membrane value(CC-part-of Cell))

While neither the first nor the second step alone require more than the description logic fragment of OWL, in conjunction they result in an OWL-Full [21] ontology.

For an adequate integration of canonical and phenotype ontologies, nonmonotonically treated formulas must be added. This requires the addition of an answer set program for each relation **CC-canonical-R **and the corresponding relations **IC-R **and **IC-lacks-R**:

IC-R(X,Y) :- ind(X),class(Y),class(Z),inst(X,Z),

   CC-canonical-R(Z,Y),

   not IC-lacks-R(X,Y).

### Additions to the OBO Relationship Ontology

The OBO Relationship Ontology [6] requires several additions for our proposal to succeed. First, the classes of **lacks **relations, as described in table 1, must be added. This will allow lacking body parts to be defined in ontologies such as the Mammalian Phenotype Ontology [10].

In the description logic variant of the Web Ontology Language [21,25] (OWL-DL), **lacks **relations can be expressed using negated statements. However, **lacks **relations are reduced to relations between individuals in a different way compared to what is done for most other relations in the OBO Relationship Ontology (cf. table 1). Ontologies developed directly in OWL-DL could use negation to avoid reference to **lacks **relations at all.

Second, **canonical-R **relations must be included as relations between categories, using the semantics introduced here. In particular, the **canonical-R **relations require a nonmonotonic knowledge representation formalism, and cannot be formalized using any form of classical logic. We presented one possible implementation using answer set semantics, but there are other alternatives. At its core, however, the definition of the **canonical-R **relations remains the same in all possible formalisms dealing with defaults: *if it is consistent to assume that *some relation holds, this relation holds.

### Use case: Integration of Mouse Anatomy and Mammalian Phenotype Ontology

The method we propose can be used in conjunction with existing tools and ontologies. Little effort is required to modify current ontologies to fit within our proposed methodology. Below, we demonstrate how to re-interpret the Adult Mouse Anatomy Ontology [1] (MA) and the Mammalian Phenotype Ontology [10] (MP) to fit within our proposed framework, and discuss problems with the current formalization in the MP.

#### Mouse Anatomy

The Adult Mouse Anatomy Ontology (MA) uses two relationships, **is-a **and **part-of**. We introduce one new relationship to the MA, which we call **canonical-has-part**, and automatically *add *for each statement of the type

*X ***part-of ***Y *(6)

the new statement

*Y ***canonical-has-part ***X*. (7)

We believe that this will result in most cases in correctly interpreted default rules, but this method will generate some inadequate statements. Therefore, manual verification will be necessary. In addition, some of the generated statements may not contain default rules, but are universally true, while some of the currently present statements involving **part-of **may not be universally true, but represent default rules. Therefore, automatically generating default rules from existing statements can only be the first step, and in the continued development of the MA, a distinction must be made between default rules and universally true statements. This may make it necessary to include additional relationships between categories in the MA, e.g. **canonical-part-of **and **has-part**.

#### Mammalian Phenotype Ontology

The Mammalian Phenotype Ontology (MP) defines, among others, categories labelled by terms such as *absent-X*. In these terms, the hidden negation must be made explicit. The MP is available in two versions, one containing only **is-a **relations, and another experimental version that attempts to define terms using relationships such as **inheres-in **[17] and categories from PATO [37] (an ontology of phenotypic properties), MA and others. The MP provides property concepts such as *absent_tail*, although we believe that it would be more adequate to term this property *absence of a tail*, because *absent_tail *suggests a reading as an object concept, namely as a tail which is absent. These properties can be composed with object concepts, e.g. *adult mouse*, in order to refer to more specific object concepts like an *adult mouse without a tail *(without explicating all of these in advance). Formally, the category *absent_tail *is defined as the intersection of *PATO:lacking physical parts*, **inheres-in ***MA:adult mouse*, and **towards ***MA:tail*. The translation to OWL [36] yields

EquivalentClasses(absent_tail

   intersectionOf(

      PATO:lacking_physical_parts

      restriction(inheres-in someValuesFrom(MA:adult_mouse))

      restriction(towards someValuesFrom(MA:tail))))

Such formalization has the problem that a reduction of *absent_tail *to relationships between individuals is inappropriate. It becomes manifest in restriction(towards someValuesFrom(MA:tail)), which enforces the existence of an instance of *MA:tail *in the OWL model [38] – yet, ontologically, there is no instance of tail if the mouse does not have a tail. If "absent tail" is taken literally, i.e., a "virtual" tail with a property of "absence" is accepted in the OWL model, the immediate objection is weakened. However, this would either imply that the **towards **link points to an arbitrary tail of some other entity, or that the mouse with the absent tail does have a tail (which may have the propery of being "absent"). This causes at the very least some inconveniences, e.g. when querying such a model for tails, one must pay attention to exclude all virtual tails. We expect that a general application of this approach to many object classes cannot be controlled with reasonable effort. The underlying problem here is a different kind of concept composition compared to that which is commonly employed in description logics and the semantics of the OBO file format [36]. For composing the concept *absent_tail *with recourse to tail, a direct link from *absent_tail *to the OWL class *MA:tail *would be required instead of a link to *instances *of the class *MA:tail*. However, links on the class level are not available if the formalization is supposed to adhere to the decidable description logic variant of OWL.

Other problems occur if the lack of a certain part is considered with respect to (sub-)parts of it. For example, a caudal vertebra is a part of a tail. The logical definition for *absent caudal vertebra *can be formulated similarly to the one for the *absent_tail*. Then the question arises to which entity the PATO property **inheres-in **should link. If *tail *is chosen, this definition cannot be applied to mice lacking a tail, because then there is nothing to inhere in. Another option is to link to mouse instead. In both cases, whenever a mouse lacks a tail as part, it also lacks all parts of the tail as part. Since each instance of *caudal vertebra *which is a part of a mouse is part of its tail, a mouse without tail lacks a *caudal vertebra*. This conclusion cannot be drawn in the approach currently taken by the MP. Moreover, we do not want to conclude that a mouse has a *caudal vertebra *as part when it lacks a *tail*.

Therefore, our suggestion is to introduce the relationship **lacks-part **and to define terms of the type *absent_X *as standing in the **lacks-part **relationship to *X*. Then, *absent_X *terms refer to categories of objects instead of properties, and those categories can be viewed as a reification of the binary **lacks-part **relation. For example, *absent_tail *would be defined as

[Term]

id: MP:0003456

name: absent tail

relationship: lacks_part MA:0000008 ! tail

By design, this use of **lacks-part **does not have the problem of non-existent instances. It also does not permit the conclusion that a mouse has a *caudal vertebra *as part when a mouse lacks a *tail*.

## Discussion

Meaningful integration of the numerous biomedical ontologies is a major task with many challenges. Currently, the infrastructure for such integration is developed in the form of top-level ontologies, biomedical core ontologies and logic-based inference systems.

### Concept conversion

The formalism we introduced requires reformulating the definitions for the categories expressed in phenotype ontologies. Categories in the form "absent-X" should be defined by, e.g., **CC-lacks-part ***X*, where *X *is a category in some canonical ontology. In some cases, this conversion can be done automatically using simple pattern matches. The Mammalian Phenotype Ontology [10] contains 395 categories of the type "absent-X", which indicate a **CC-lacks-part **relationship. However, it is likely that an amount of manual curation will be required to convert relevant concepts into the required form. We believe that the advantages gained by having a common framework for integrating a large number of biomedical ontologies justifies this effort, in particular since it also allows for a semantically richer definition of terms.

### Defaults and canonical knowledge

We introduce the notion of "default knowledge" as a technical term within the formalism we propose. We do not discuss what a "default" is, or when a piece of knowledge becomes a default, in contrast to merely contingent knowledge. Developers of domain ontologies must decide this. Widespread acceptance of some fact, its sanction by scientific discourse, or its implicit use in scientific writing may provide starting points for finding defaults. These principles have been used to construct the Foundational Model of Anatomy [9] (FMA). The Mammalian Phenotype Ontology classifies categories almost exclusively under categories named "abnormal-X". The ontology of phenotypic properties (PATO) contains the property of being "abnormal". Each of the corresponding categories and annotations can be investigated and the corresponding default rule identified. Not all pieces of information contained in ontologies such as the FMA will be default knowledge, but we expect that a significant number of facts can be translated to the formalisms we propose, thereby making the nature of the fact as a default explicit.

There is a difference between canonical and default knowledge, in particular in the context of anatomy. Canonical human anatomy, for example, describes an idealized, prototypical human being. This does not necessarily coincide with a normal human being, i.e. in the sense of statistically averaged values. Defaults, on the other hand, tend to capture in their commonsense usage the *normal *cases of a category. We believe that the framework of default logic, compared with other systems, provides the most adequate interpretation for canonical knowledge. However, while certainly needed, a precise distinction between normal, default and canonical knowledge is out of the scope of this study.

### Comparison with other approaches

The important role of accommodating exceptions and defaults in biomedical knowledge representation has been recognized previously [39], where patterns to deal with a variety of cases were introduced and discussed. These cases are based on the description logic fragment of OWL [21], and therefore monotonic logic. In [39], three types of exceptions that occur in biomedical knowledge bases are distinguished:

1. Single exceptions: "Arteries carry oxygenated blood" except for the pulmonary artery. In [39], it is proposed to reformulate this statement to "Arteries except the pulmonary artery carry oxygenated blood".

2. Exceptions due to context: "The normal human manus has five digits", with "human" and "normal" being treated as explicit contexts.

3. Unpredictable number of exceptions, exceptions from exceptions, etc., such as drug uses, contraindications and interactions.

We offer a method for representing these types of exceptions using a nonmonotonic knowledge representation formalism. We use answer set programs to provide the semantics for treating knowledge in OWL as default knowledge with additional exceptions. This does not exclude the possibility to treat these types of exceptions exclusively in a monotonic logic such as OWL where appropriate, for which [39] provides a solution. The solution in [39] to the example of arteries carrying oxygenated blood, except the pulmonary artery, has the problem that it must be explicitly known that some artery is *not *the pulmonary artery, in order to conclude that this artery carries oxygenated blood. There may be cases where this is not wanted, especially if the exception occurs very rarely. In particular, if there is only one rare exception to a rule and some statement influencing the property which changes with this exception is asserted, then the knowledge engineer will usually make this exception explicit, and ignore it otherwise. Then, a question whether an artery carries oxygenated blood evaluates to true, except when it is *proven *that this artery is the pulmonary artery. On the other hand, the solution proposed by [39] is guaranteed to provide the correct inference in every case. Depending on the users and uses of a knowledge base or ontology, different representations for this case may be selected, and in many cases the treatment in [39] is adequate.

Case two is solved by explicitly introducing a context argument, in the form of additional properties, e.g., by introducing some relation *hasAnatomicalStatus *which maps to "normal". Then, a *Mouse *that has an anatomical status "normal" could have, e.g., a tail and a head as part. If a mouse had no tail, it can be concluded that it is an anatomically abnormal mouse. However, then it would be impossible to conclude that it still has a head. An extension to the solution in [39] would be to make the context more fine-grained, by specifying mouse with anatomically normal tails, heads, and so on. This comes down to specifying an enormous number of exceptions in a monotonic logic, and in order to obtain a correct answer to a query for all the parts of some individual mouse, all these exceptions must be explicitly excluded. It would not be possible to simply state that some entity is a mouse in order to obtain its parts. Instead it is required to specify explicitly which parts are normal and abnormal, which means in essence to add the answers to the query asked.

The third case in [39] is closest in spirit to our work, as one of the proposals is to use a hybrid reasoning system in order to deal with it. We have extended this idea by giving a formal account of our treatment of exceptions, which is based on a well-studied nonmonotonic logic, and is implemented in a computationally tractable framework. It can also be used in conjunction with appropriate upper ontologies. Further, we have shown how to use this formalism to achieve interoperability between canonical and phenotype ontologies in biology. And finally, we give an implementation of our ontology and support for reasoning over exceptions. This could be achieved because recent years have seen an increasing effort in developing reasoners for the Semantic Web and extending them in various ways, among them the implementation we are using, DLVHEX.

We believe that our solution to the problem of exceptions and deviations from a canonical ontology is more general than the proposal in [39]. In our opinion, the knowledge contained in a canonical ontology is inherently default knowledge. There is no adequate solution for representing this type of knowledge in a monotonic knowledge representation formalism. Representation in monotonic logic requires exceptions to be encoded in the ontology either as a list of exceptions to an axiom, or using a general "abnormality" predicate. For example, the fact that mice usually have some tail as part can be represented as "Mouse **has-part **Tail except when ..." followed by a complete list of exceptions. Alternatively, "Mouse" can be replaced by "normal mouse" in the rule, and a mouse without a tail is not normal. The first solution requires complete knowledge of all known exceptions. These must additionally be explicitly excluded in every query for parts of the mouse. The second way does not require this knowledge of exceptions, but allows for no further inferences once a mouse is known to be not normal. Defaults and exceptions cannot be dealt with in a monotonic logic without substantially modifying the canonical ontology, and limiting the ability to query the ontology.

### Limitations

A major drawback of the system we are using, DLVHEX, is its use of RACER [40] as a description logic reasoner and of DLV [33] as a datalog system. RACER and DLV are proprietary software. In order to be of general use and high quality, an implementation entirely based on free software is beneficial, if not necessary [41,42].

A number of formalisms have been proposed as a solution to handling defaults in Semantic Web representation languages or other knowledge representation formalisms. Many require modifying the language, and therefore changing tools that are used to develop ontologies. Many biomedical ontologies are developed using tools such as OBO-Edit [43] by biology experts, but not necessarily experts in logic or formal ontology. The solution we propose requires no changes to existing tools, since we are using a hybrid reasoning mechanism. Tools that are currently in use can be used further by the ontology developers. The additional semantic features that allow for the treatment of canonical relations as defaults are maintained separately from the ontologies in which they are used.

## Conclusion

In this paper we tackle the problem of integrating biomedical ontologies to facilitate interoperability among them and thus among information systems based on them. We particularly focus on adequately treating two kinds of ontologies, namely canonical and phenotype ontologies, e.g., the Mouse Anatomy ontology and the Mammalian Phenotype ontology. Given this distinction, we have argued that canonical ontologies represent default knowledge. Their integration with ontologies covering phenotypes may thus lead to inconsistencies if used within a classical logic framework, because some phenotypic descriptions are exceptions to defaults. We have shown how existing techniques from knowledge representation can be used to resolve these problems. Moreover, our solution uses the biological core ontology GFO-Bio as an ontological foundation, which provides support for our solution through higher order categories and relations. Integrating canonical and phenotype ontologies, however, requires both an appropriate ontological basis as well as a nonmonotonic representation formalism.

Our work primarily extends the OBO Relationship Ontology [6], and requires few changes to domain ontologies. In particular, our proposal does not require modifications in the tools that domain ontology developers use for curating ontologies, or changes in the way these ontologies are developed and stored. Our solution remains fully compatible with the OBO representation format, and addresses all logical, formal and computational requirements in our proposed extension to the OBO Relationship Ontology. It is there that nonmonotonic semantics must be made available to users. In its current form, based on a classical, monotonic logic, the OBO Relationship Ontology cannot support interoperability between all ontologies that will become part of the OBO Foundry, in particular between anatomy and phenotype ontologies. Our proposal aims to bring about effective interoperability and integration between the ontologies in the OBO Foundry without the need to modify the representation formalism or the tools used in ontology curation and analysis.

## Availability and Requirements

Project name: GFO-Bio/NMR

Project homepage: 

Operating systems: GFO-Bio and axiomatization are platform independent, reasoning using DLVHEX requires GNU/Linux or Mac OS X.

Programming languages: OWL, Semantic Web Rule Langue (SWRL), datalog, Java Other requirements: Parts of our implementation require DLVHEX, DLV and RACER.

License: Modified BSD License. DLVHEX requires RACER and DLV. Both are proprietary software.

## Competing interests

The authors declare that there are no competing interests.

## Authors' contributions

HH conceived the initial idea on using nonmonotonic reasoning for integrating ontologies, RH the relevance for anatomy and phenotype ontologies. HH, FL, JK and RH designed the framework for consistent integration of anatomy and phenotype ontologies. RH implemented the framework and performed the tests. All authors contributed to writing the paper, have read and approved the final version of this manuscript.
